# The Systolic and Diastolic Cardiac Function of Patients With Type 2 Diabetes Mellitus: An Evaluation of Left Ventricular Strain and Torsion Using Conventional and Speckle Tracking Echocardiography

**DOI:** 10.3389/fphys.2021.726719

**Published:** 2022-01-07

**Authors:** Qing-mei Yang, Jian-xiu Fang, Xiao-yan Chen, Hong Lv, Chun-song Kang

**Affiliations:** ^1^Department of Ultrasound, Shanxi Bethune Hospital, Shanxi Academy of Medical Sciences, Tongji Shanxi Hospital, Third Hospital of Shanxi Medical University, Taiyuan, China; ^2^Department of Ultrasound, Tongji Hospital, Tongji Medical College, Huazhong University of Science and Technology, Wuhan, China

**Keywords:** 2D-speckle tracking imaging, strain, diabetic cardiomyopathy, diabetes mellitus, left ventricular

## Abstract

**Objectives:** This study aimed to quantify left ventricular (LV) myocardial strain and torsion in patients with type 2 diabetes mellitus (T2DM) and evaluate their systolic and diastolic function using conventional and speckle tracking echocardiography.

**Methods:** Forty-seven patients with T2DM were divided into a group without microvascular complications (the DM A group) and a group with microvascular complications (the DM B group), while another 27 healthy participants acted as the control group. All the participants had had an echocardiography examination. All the original data were imported into EchoPAC workstation for the analysis and quantification of LV strain and torsion.

**Results:** Compared with the control group, the LV end-diastolic volume, end-systolic volume, and ejection fraction of the DM A and DM B groups showed no significant differences, but the global longitudinal strain and the global circular strain were reduced in the DM B group. There were significant differences in the left ventricular relative wall thickness (RWT), left ventricular mass index (LVMI), the early mitral valvular blood flow velocity peak/left ventricular sidewall mitral annulus late peak velocity, left ventricular sidewall mitral annulus early peak velocity/left ventricular sidewall mitral annulus late peak velocity, isovolumic relaxation time, peak twisting, peak untwisting velocity (PUV), untwisting rate (UntwR), time peak twisting velocity (TPTV), and time peak untwisting velocity (TPUV) between the DM A, DM B, and control groups. While the peak twisting velocity (PTV) was slower in the DM B group compared with the control group, the RWT, PTV, PUV, UntwR, TPTV, and TPUV in the DM B group were significantly different from the DM A group.

**Conclusion:** The cardiac function of patients with T2DM in its early stages, when there are no microvascular complications, could be monitored with the analysis of two-dimensional strain and torsion.

## Introduction

As diabetes mellitus (DM) develops there is an evident increase in cardiovascular mortality (Rossi and American Diabetes Association, 2018). It is known that DM can lead to diabetic cardiomyopathy, defined as myocardial dysfunction, independent of coronary artery disease, and hypertension (Lee and Kim, 2017; Jia et al., 2018). Diastolic dysfunction is a major characteristic of early diabetic cardiomyopathy, while systolic dysfunction is a later development (Boudina and Abel, 2007; Bergner and Goldberger, 2010). It is clear that DM with diabetes cardiomyopathy will have a more adverse clinical outcome (Voors and van der Horst, 2011), and so the early detection of diabetic heart disease is of paramount importance because timely lifestyle modifications and medical interventions may prevent or delay the subsequent development of heart failure (Ghio et al., 2001; Fang et al., 2005).

Two-dimensional speckle tracking echocardiography (2D-STE) is a novel method for the angle-independent quantification of LV strain, strain rate, and twist, with good reproducibility (Notomi et al., 2005; Qu et al., 2007; Blessberger and Binder, 2010; Mor-Avi et al., 2011; Huang et al., 2018), and STE can quantitatively analyze cardiac diastolic and systolic function (Santos-Gallego et al., 2020).

This study aims to quantify the LV myocardial strain and torsion of patients with type 2 diabetes mellitus (T2DM) and evaluate the systolic and diastolic function by using conventional and speckle tracking echocardiography to determine the changes in the heart function of patients in the early stages of T2DM without microvascular complications.

## Materials and Methods

### Subjects

Between December 2017 and July 2018, patients with T2DM were recruited for this study. The diagnosis of T2DM was made in line with the 2010 American Diabetes Association guidelines (American Diabetes Association, 2012). The participants were divided into a group without microvascular complications (the DM A group) and a group with microvascular complications (the DM B group). Having microvascular complications meant the patient had been diagnosed as having at least one of the following complications: diabetic retinopathy, diabetic nephropathy, or diabetic peripheral neuropathy. Healthy participants (their physical examination, electrocardiogram, and echocardiography tests were normal) were used as the control group. In Group B, there are seven cases with fundus lesion, one case with diabetic nephropathy (GRF glomerular filtration rate = 64 mmol/min), nine cases with peripheral neuropathy (five cases with nerve threshold decrease and four cases with threshold disappearance in). Peripheral vascular lesion was detected in four cases (three cases of carotid intima thickening, and one case of carotid artery plaque formation with the size 9.3*1.6 mm). Among them, one case underwent multiple complications with peripheral nerves, blood vessels and fundus lesions at the same time. All the subjects underwent echocardiography, electrocardiography, and biochemical examinations. This study was conducted in accordance with the Declaration of Helsinki and approved by the ethics committee of our hospital. All the participants signed informed consent.

### Inclusion and Exclusion Criteria

The inclusion criteria were as follows: (1) patients who were diagnosed as having T2DM; (2) patients older than 18 years of age; and (3) patients who had signed informed consent. The exclusion criteria were as follows: (1) patients who had hypertension (systolic blood pressure ≥ 140 mmHg); (2) patients who had coronary heart disease, myocardial infarction, heart valve disease, cardiomyopathy, or congenital heart disease; (3) patients who had severe arrhythmia and other diseases that affect heart function; and (4) patients whose data were incomplete.

### Echocardiography

Echocardiographic imaging was performed using a Vivid E9 GE Medical Systems commercial scanner (1–5 MHz, 5S probe). The subjects were lying on their left side and breathing calmly. Electrocardiograms were recorded simultaneously during the examinations.

Using 2D echocardiography with a 5S probe, the early and late mitral valvular blood flow velocity peak (E and A, respectively) and left ventricular sidewall mitral annulus early and late peak velocity (e and a, respectively) were obtained, and E/A, e/a, and E/e were calculated. The left ventricular end-diastolic volume (LVEDV), end-systolic volume (LVESV), and ejection fraction (LVEF %) were measured using the Simpson biplane method, and the relative wall thickness (RWT) and left ventricular mass index (LVMI) were calculated. The cardiac isovolumic contraction time (IVCT) and isovolumic relaxation time (IVRT) were also obtained. The quality of the image was then adjusted to make the endocardial display clear, and the patient was instructed to hold their breath after a deep breath, and the apical four-, three-, two-chamber and short-axis views (frame rates, 75–128/s) of three consecutive cycles were stored for offline analysis.

### Echocardiographic Image Analysis

The apical four-, three- and two-chamber views were analyzed using 2D-STE software (2D-Strain, EchoPAC PC113, GE Healthcare). Points are placed on the apical long-axis at the intersection of the mitral valve annulus and the apical position, and on the top, bottom, left, and right of the short-axis section. The software automatically calculates the 2D myocardial strain of the left ventricle in 18 segments. After manual fine-tuning, spot tracking data can be obtained that generate LV apex series long-axis cut planes, the sections of each wall, the global longitudinal strain (GLS), and LV short-axis planes. Strain data, namely the global radial strain (GRS) and global circular strain (GCS), as well as peak twisting (Ptw) data, namely the peak twisting velocity (PTV), peak untwisting velocity (PUV), time peak twisting velocity (TPTV), and time peak untwisting velocity (TPUV) were recorded, and the untwisting rate (UntwR) was calculated using the formula UntwR = (peak of systolic twisting − twisting when the mitral valve opens)/peak twisting/IVRT × 100%.

### Intra- and Inter-observer Reproducibility

Intra- and inter-observer variability for GLS and Ptw were determined by repeating measurements in all the T2DM patients and normal subjects. The intra-observer reliability assessment was performed 1 week apart by the same observer on the same echocardiographic images in a random order, while the assessment of inter-observer reproducibility was carried out by another independent observer.

### Statistical Analysis

All statistical analyses were performed using the SPSS 17.0 software package (SPSS Inc., Chicago, IL). The continuous variables of normal distribution were expressed as mean ± standard deviation, the continuous variables of non-normal distribution were expressed as median (interquartile range), and the categorical variables were expressed as a frequency [percentage (%)]. For multiple comparisons, each value was compared using a one-way analysis of variance followed by a Dunnett’s test, when each datum conformed to normal distribution, while the non-normally distributed continuous data were compared using nonparametric tests. The count data were tested using the Chi-square test, and Pearson’s correlation was chosen to the test correlations between the parameters of the three groups. A value of *p* < 0.05 was considered statistically significant.

The intraclass correlation coefficient (ICC) was used to evaluate inter- and intra-observer variability. Clinical significance was categorized as follows: good, ICC ≥ 0.75; moderate, ICC ≥ 0.4 and < 0.75; and, poor, ICC < 0.4.

## Results

### General Characteristics

A total of 47 patients with T2DM were included in this study, comprising 32 males and 15 females. These 47 patients were divided into a group without microvascular complications (the DM A group) with 28 cases, and a group with microvascular complications (the DM B group) with 19 cases. Another 27 healthy participants were recruited as the control group. Among the three groups, no statistical differences were found in gender (*p* = 0.889), age (*p* = 0.672), heart rate (*p* = 0.819), body mass index (*p* = 0.668), heart rate (*p* = 0.819), EF (*p* = 0.118), E/A ratio (*p* = 0.489), and IVCT (*p* = 0.404). The total cholesterol and high-density lipoprotein levels were higher in patients with T2DM than in the control group. However, the low-density lipoprotein levels were lower in patients with T2DM than in the control group. In addition, hemoglobin A1c levels were higher in the DM B group than in the DM A group. The RWT and LVMI in the DM group were higher than in the control group (*p* = 0.023 and *p* = 0.045, respectively). The e/a ratio (*p* = 0.014) in the DM groups was lower than in the control group while the E/e ratio (*p* = 0.042) and the IVRT (*p* = 0.040) were higher. However, there were no statistical differences in the E/A ratio (*p* = 0.489) and the IVCT (*p* = 0.404) between the three groups (*p* > 0.05). There were no statistical differences in the LVEDV (*p* = 0.439) and LVESV (*p* = 0.744) between the control group and the DM A group, but the LVEDV (*p* = 0.011) and LVESV (*p* = 0.010) were higher in the DM B group than in the DM A group.

The basic information and conventional echocardiography results of the three groups are listed in Tables 1 and 2.

**Table 1:** Basic information of the three groups.

Variable	Control group	DM A group	DM B group	*F/χ^2^*	*p*
Gender (male/female)	18/9	19/9	13/6	0.235	0.889
Age	49.93 ± 8.28	51.42 ± 8.94	52.16 ± 9.22	0.399	0.672
Heart rate (/min)	70.73 ± 8.69	72.24 ± 11.76	70.27 ± 11.16	0.201	0.819
BMI (kg/m^2^)	23.05 ± 1.99	23.56 ± 2.49	23.39 ± 1.49	0.406	0.668
Diabetes duration (years)	—	9.11 ± 5.19	10.36 ± 3.45	0.701	0.566
Fasting plasma glucose (mmol/L)	4.63 ± 1.12	8.14 ± 0.91^*^	8.37 ± 0.69^*^	15.709	0.000
HbA1c (%)	—	6.08 ± 0.36	9.22 ± 0.92^#^	19.402	0.000
Total cholesterol (mmol/L)	4.13 ± 0.58	4.41 ± 0.58^*^	4.47 ± 0.38^*^	3.317	0.037
Plasma triglycerides (mmol/L)	1.19 ± 0.27	1.89 ± 0.44^*^	1.81 ± 0.67^*^	33.210	0.000
LDL (mmol/L)	2.78 ± 0.30	2.25 ± 0.60^*^	2.34 ± 0.51^*^	14.675	0.00
HDL (mmol/L)	1.21 ± 0.83	1.30 ± 0.64	1.33 ± 0.75	0.700	0.502

Data given as the mean ± SD. HDL, high-density lipoprotein cholesterol; LDL, low-density lipoprotein cholesterol.

* *p < 0.05 vs. normal group*.

# *p < 0.05 vs. DM A group*.

**Table 2:** Echocardiographic parameters of the three groups.

Variable	Control group	95% CI	DM A group	95% CI	DM B group	95% CI	*F/χ^2^*	*p*
RWT	0.42 ± 0.08	0.35–0.48	0.47 ± 0.07^*^	0.42–0.53	0.44 ± 0.07^*^^,^^#^	0.39–0.49	7.139	0.003
LVMI (g/m^2^)	86.59 ± 15.65	73.44–97.79	101.56 ± 14.25^*^	86.94–108.02	107.49 ± 17.89^*^	88.69–113.96	3.401	0.045
EDV (ml)	83.78 ± 14.19	78.17–89.39	79.42 ± 17.74	72.91–85.93	95.53 ± 32.35^#^	79.93–112.12	3.435	0.037
ESV (ml)	34.2 ± 6.35	31.79–36.81	33.13 ± 8.0	30.19–36.07	36.13 ± 14.0^*^^,^^#^	31.96–55.30	3.936	0.024
EF	59.07 ± 2.32	58.16–60.09	58.39 ± 2.65	57.41–59.99	56.53 ± 7.10	53.10–59.95	2.201	0.118
E/A	0.99 ± 0.36	0.85–1.13	0.94 ± 0.25	0.84–1.03	0.95 ± 0.32	0.79–1.10	0.257	0.774
e/a	0.88 ± 0.36	0.89–1.03	0.69 ± 0.14^*^	0.74–1.02	0.67 ± 0.18^*^	0.64–0.74	6.072	0.004
E/e	8.49 ± 1.67	7.84–9.15	10.14 ± 2.02^*^	9.40–10.89	14.9 ± 10.37^*^	9.90–19.90	8.273	0.001
IVRT (ms)	77.11 ± 9.99	79.81–90.11	86.14 ± 14.38^*^	76.82–88.85	86.74 ± 13.35^*^	73.67–83.80	1.115	0.333
IVCT (ms)	62.19 ± 17.71	55.18–69.19	58.65 ± 14.55	53.31–63.98	62.68 ± 17.79	54.11–71.26	0.481	0.620

Data given as the mean ± SD. RWT, relative wall thickness; LVMI, left ventricular mass index; IVCT, isovolumic contraction time; IVRT, isovolumic relaxation time.

* *p < 0.05 vs. normal group*.

# *p < 0.05 vs. DM A group*.

### Myocardial 2D Strain and Torsion Parameters

Compared with the control group, the GLS in the DM A (*p* = 0.006) and the DM B group (*p* = 0.004) were reduced, and the GCS in the DM B group was reduced (*p* = 0.038); the GRS in the DM A group and the DM B group showed a trend of gradual reduction. The GLS (*p* = 0.046) in the DM B group was lower than in the DM A group, as shown in Table 3.

**Table 3:** 2D strain and torsion parameters in the three groups.

Variable	Control group	95% CI	DM A group	95% CI	DM B group	95% CI	*F*	*P*
GRS (%)	40.27 ± 11.8	35.56–44.93	37.41 ± 12.61	33.35–41.95	34.05 ± 7.67	33.10–39.36	0.812	0.448
GCS (%)	−22.1 ± 3.02	−23.29–(−20.90)	−20.94 ± 3.96	−22.39–(−19.51)	−19.57 ± 3.16^*^	−21.94–(−17.38)	2.868	0.063
GLS (%)	−20.23 ± 2.45	−20.79–(−17.48)	−17.25 ± 2.43^**^	−19.15–(−15.56)	−15.1 ± 3.22^**^^,^^#^	−20.24–(−15.25)	3.256	0.044
Ptw (°)	18.33 ± 3.97	15.72–19.74	15.04 ± 4.77^*^	14.33–18.89	13.93 ± 2.83^*^	12.47–17.84	1.142	0.325
TPTV (ms)	222.44 ± 44.64	204.78–24.20	181.09 ± 57.01^*^	167.88–207.86	161.60 ± 37.91^**^^,^^#^	133.83–180.80	9.749	0.000
PTV (%)	107.53 ± 30.86	95.32–119.73	105.00 ± 29.89	93.37–115.94	87.04 ± 21.15^*^^,^^#^	77.06–105.55	3.122	0.048
PUV (%)	−117.58 ± 30.07	−141.33–(−94.60)	−102.49 ± 49.65^*^	−130.54–(−84.01)	−96.26 ± 31.25^*^^,^^#^	−122.68–(−90.46)	3.337	0.041
TPUV (ms)	418.30 ± 38.36	403.54–433.87	431.14 ± 39.16^*^	422.06–462.26	461.7 ± 35.28^*^^,^^#^	425.38–469.47	3.606	0.038
UntwR (%)	0.54 ± 0.19	0.47–0.62	0.41 ± 0.16^*^	0.33–0.54	0.32 ± 0.20^**^^,^^#^	0.19–0.45	4.662	0.035

Data given as the mean ± SD. GLS, global longitudinal strain; GRS, global radial strain; GCS, global circular strain; Ptw, peak twisting; PTV, peak twisting velocity; PUV, peak untwisting velocity; TPTV, time peak twisting velocity; TPUV, time peak untwisting velocity; UntwR, untwisting rate.

* *p < 0.05 vs. normal group*.

** *p < 0.01 vs. control group*.

# *p < 0.05 vs. DM A group*.

**Figure 1:**
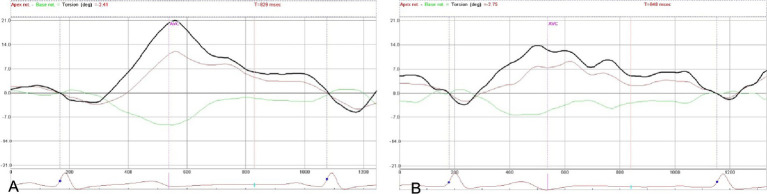
The twisting and untwisting curves: **(A)** is the control group, and **(B)** is the DM groups.

The curves in the DM A group, the DM B group, and the control group showed similar trends, as shown in Figure 1. Compared with the control group, in the DM A and DM B group, the LV Ptw (*p* = 0.045 and *p* = 0.013), PUV (*p* = 0.03 and *p* = 0.039), and UntwR (*p* = 0.028 and *p* = 0.009) were reduced, and the TPTV (*p* = 0.010 and *p* = 0.000) was lower. However, the TPUV (*p* = 0.048) was higher, while the PTV (*p* = 0.040) in the DM B group was also lower compared with the control group. Compared with the DM A group, the PTV (*p* = 0.023), PUV (*p* = 0.047), and UntwR (*p* = 0.039) in the DM B group were lower, and the TPTV (*p* = 0.039) was lower, while the TPUV (*p* = 0.048) was higher, as shown in Table 3.

### An Analysis of the Parameters Showing Diastolic Dysfunction

**Figure 2:**
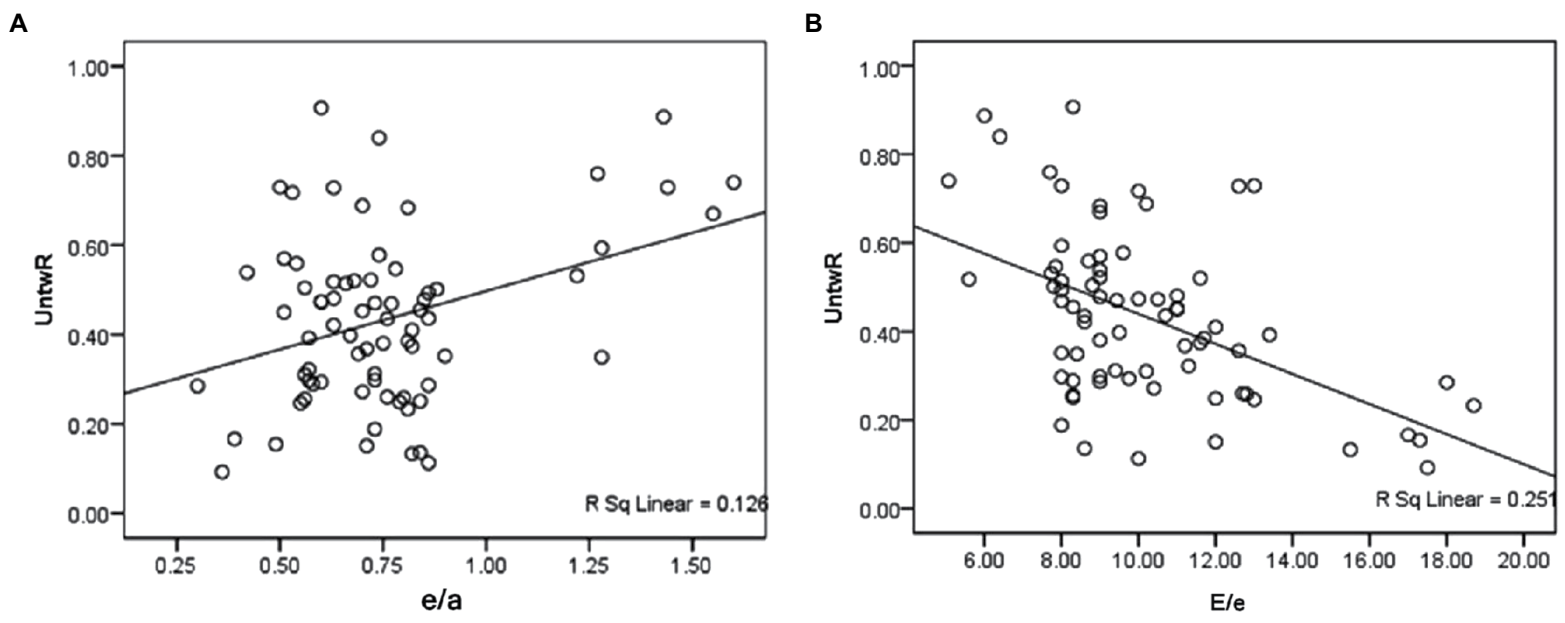
**(A)** Shows that the UntwR was positively correlated with e/a. **(B)** Shows that the UntwR was negatively correlated with E/e.

The general strain and twisting parameters were analyzed using Pearson correlation analysis between two variables to compile a new index for assessing LV dysfunction. The confounding factors consisted of the E/A, e/a, and E/e ratios. The inclusion of these parameters was based on the results of the above data. As shown in Table 4 and Figure 2, the UntwR was positively correlated with e/a (*r* = 0.37 and *p* = 0.002), and negatively correlated with E/e (*r* = −0.5 and *p* = 0.000). The results demonstrate that UntwR reduced with the decrease of left ventricular diastolic function, and, thus, it may be a parameter that can be used to evaluate diastolic dysfunction.

**Table 4:** Correlation analysis between general parameters and strain parameters.

	Ptw		TPTV		PTV		PUV		TPUV		UntwR	
	*r*	*p*	*r*	*p*	*r*	*p*	*r*	*p*	*r*	*p*	*r*	*p*
E/A	0.138	0.230	0.101	0.381	0.091	0.430	0.010	0.931	0.048	0.678	0.244	0.320
e/a	0.056	0.629	0.303	0.071	−0.056	0.627	0.080	0.488	0.002	0.986	0.370^*^	0.002
E/e	0.072	0.532	−0.209	0.068	0.063	0.586	−0.050	0.668	0.080	0.487	−0.502^**^	0.000

Ptw, peak twisting; PTV, peak twisting velocity; PUV, peak untwisting velocity; TPTV, time peak twisting velocity; TPUV, time peak untwisting velocity; UntwR, untwisting rate.

* *p < 0.05;*;

** *p < 0.01*.

### Inter-observer and Intra-observer Variability

The results for the intra- and inter-observer variability for the GLS and Ptw repeated measurements in all the subjects are shown in Table 3. The ICCs for inter- and intra-observer variability were 0.847–0.935 and 0.812–0.907, respectively, which indicates that the software analysis used in the study had good repeatability and reliability.

## Discussion

This study found that the 2D myocardial strain parameters, particularly the GLS, as well as the twisting parameters of the DM B group, were reduced when compared with the DM A group and the normal control group. Thus, UntwR may be a parameter that can evaluate diastolic dysfunction.

Left ventricular ejection fraction is always used for detecting contractile function in normal subjects and patients suffering from cardiac disease. However, in this study, no significant difference in the LVEF between the T2DM patients and healthy individuals was found. There were no differences in the LVEDV and LVESV between the control group and the DM A group, but they were higher in the DM B group than in the DM A group. It is suggested that diabetes mellitus may have little effect on LVEDV and LVESV, while microvascular disease is the larger influencing factor. The possible reasons for these results are as follows: (1) the condition of the DM A group was mild, and so cardiac structure had not yet changed significantly; (2) conventional echocardiographic parameters cannot reflect early systolic dysfunction in T2DM patients; and (3) the sample size was small. The RWT and LVMI in the DM group were higher than in the control group, may be related to pathological changes such as myocardial cell hypertrophy, necrosis and increase in myocardial weight, caused by the continuous and progressive damage of hyperglycemia to the myocardium, the accumulation of lipids, and the subsequent disorder of autonomic nerve and humoral regulation (Rijzewijk et al., 2008; van den Bergh et al., 2008).

In order to find the effect of structural measures on STE, we explored the relationship between structural measures (RWT, EDV, and LVMI) and STE measures (GLS, GCS, GRS, and UntwR) using multiple regression analysis (see Supplementary Table S1). We found that only LVMI had some effect on GCS. There was no difference in GCS between DM A group and DM B group, indicating that microvascular disease could not affect GCS. Therefore, according to the results of multiple linear regression analysis, GCS was affected by LIMI, which explained 9.3% of its variation. While GRS, GLS, and UntwR were not related to structural measures such as RWT, EDV, and LVMI. Meanwhile, GLS showed significant difference between DM A group and DM B group, indicating that GLS may be mainly affected by microvascular disease. UntwR showed significant difference between DM A group and DM B group, indicating that UntwR may be mainly affected by microvascular disease. Considering that GLS, GRS, and GCS were used to evaluate LV global systolic function and UntwR was related to diastolic function, we inferred that both structural differences and microvascular disease could affect systolic function, while diastolic function may be mainly affected by microvascular disease.

In addition, it was found that the 2D myocardial strain parameters, especially the GLS, showed a decreasing trend with the development of DM. This indicates that as DM progresses, the occurrence of related microvascular complications increases, and the longitudinal myocardial contractile function becomes even more abnormal. This may happen because GLS is related to endocardial myocardial contraction and is more sensitive to myocardial fibrosis and myocardial perfusion insufficiency (Hansen et al., 2002; Nakai et al., 2009; Ernande et al., 2011). With the occurrence of microvascular complications, myocardial damage can be serious and even lead to cell necrosis or myocardial ischemia, for example, and, at this point, there will be myocardial stiffness and ventricular remodeling. These pathological changes (Rijzewijk et al., 2008; van den Bergh et al., 2008) and the changing trend of the strain parameters were basically similar to the findings of previous studies (Ernande et al., 2010; Li et al., 2014; Sugimoto et al., 2017; Lin et al., 2018; Mirea et al., 2018; Wang et al., 2018). It can be concluded that strain parameters, such as GLS, are very sensitive markers of LV systolic function, much more so than LVEF (Hung et al., 2010; Park et al., 2018; Santos-Gallego et al., 2019).

The E/A ratio is used as a qualitative indicator, when an E/A ration <1 or >2 can be considered to reflect relaxation after myocardial contraction, the LV diastolic suction effect, and LV compliance, and it can be used to evaluate myocardial dysfunction. The e/a ratio can reflect the pulmonary artery incarceration pressure (Mousavi et al., 2010). When the LV diastolic function is reduced, the LV wall is stiff and the compliance is reduced, so the left atrial pressure increases, and the pulmonary artery incarceration pressure increases (Santos-Gallego et al., 2019). The e/a ratio is a parameter that is not affected enough by hemodynamics to evaluate LV diastolic function. However, the E/e ratio reflects changes in LV filling pressure and can be considered as a more sensitive parameter for evaluating changes in diastolic function. In this study, there was no significant change in the E/A ratio, maybe because the E/A ratio < 1 and the E/A ratio > 2 measurements were put together in one broad band. However, the e/a and E/e ratios were consistent with the known changes in diastolic function in patients with DM.

In this study, the PUV and UntwR, particularly the UntwR, were reduced, and the TPUV was higher. Increased oxidative stress and lipid deposition can cause myocardial stiffness and damage to endothelial cells leading to the increased production of cell contractile substances, and microangiogenic lesions can cause myocardial cells to have an insufficient blood supply, which can result in necrosis (Rijzewijk et al., 2008; van den Bergh et al., 2008). DM brings about reduced compliance and abnormal diastolic function, and with the progress of the disease, hyperglycemia stimulates the myocardium even more, and the diastolic function deteriorates even further. The changing trends in the strain parameters observed in this study are basically similar to those seen in previous studies (Chang et al., 2018; Spinelli et al., 2019; Zhang et al., 2020) and seem to confirm that untwisting parameters can reflect LV diastolic dysfunction.

Left ventricular contraction produces a counterclockwise twist. At present, it is believed that LV accumulates energy during the twisting process that allows LV to more effectively draw blood from the left atrium into the LV during diastole. However, the specific role of the twist in the myocardial contraction has yet to be clarified. In this study, the Ptw, TPTV, and PTV were reduced in T2DM, and the EF showed a decreasing trend. These changing trends in the strain parameters are similar to previous studies (Pacileo et al., 2011; Jia et al., 2020), indicating that the twisting parameters may have a certain relationship with the contractile function of LV, which can be more sensitively reflected when a slight abnormality occurs in the contractile function of DM patients.

The results also showed that UntwR was positively correlated with the e/a ratio and negatively correlated with the E/e ratio and that the correlation between UntwR and E/e was slightly stronger. UntwR refers to the velocity of untwisting in the isovolumic diastolic time, and its decrease can be considered as a manifestation of LV diastolic function reduction. LV wall compliance is reduced in DM because of myocardial lipid deposition, interstitial fibrosis, and microvascular disease, which leads to an increase in LV filling pressure, and an increased E/e ratio. The results showed that the untwisting angle in isovolumic diastole decreased and the IVRT was prolonged due to the decrease in LV wall compliance and wall stiffness, which ultimately led to the decrease in the UntwR, which was positively correlated with the negative correlation between the UntwR and the E/e ratio.

There were several limitations to this study. Firstly, it was only a single-center trial and the sample size was limited due to there being many subjects who had to be excluded in the absence of a cardiac history. Secondly, the type of diabetes complication was not investigated, and it is known that DM complications can exist alone or coexist. Thus, the impact of any DM complication on myocardial function remains unknown to some extent and needs further research. Thirdly, STE has limitations in itself, as its use is affected by the quality of the 2D images.

## Conclusion

Global longitudinal strain is reduced in the early stage of diabetes and decreases significantly with the emergence of DM microvascular complications. The systolic function may also decrease in the early stage of diabetes and will be more obvious with the advent of microvascular complications. 2D-STE could be utilized to monitor the cardiac function of patients with T2DM in its early stages, when they have not yet developed microvascular complications.

## Data Availability Statement

The raw data supporting the conclusions of this article will be made available by the authors, without undue reservation.

## Ethics Statement

This study was conducted in accordance with the Declaration of Helsinki and approved by the Ethics Committee of Shanxi Academy of Medical Sciences. The patients/participants provided their written informed consent to participate in this study.

## Author Contributions

Q-mY, J-xF, and X-yC conceived the idea and conceptualized the study. X-yC and HL collected the data and analyzed the data. Q-mY and J-xF drafted the manuscript. C-sK and J-xF reviewed the manuscript. All authors contributed to the article and approved the submitted version.

## Conflict of Interest

The authors declare that the research was conducted in the absence of any commercial or financial relationships that could be construed as a potential conflict of interest.

## Publisher’s Note

All claims expressed in this article are solely those of the authors and do not necessarily represent those of their affiliated organizations, or those of the publisher, the editors and the reviewers. Any product that may be evaluated in this article, or claim that may be made by its manufacturer, is not guaranteed or endorsed by the publisher.
