# Supplementary material for: The Systolic and Diastolic Cardiac Function of Patients With Type 2 Diabetes Mellitus: An Evaluation of Left Ventricular Strain and Torsion Using Conventional and Speckle Tracking Echocardiography
Source: Front Physiol. 2022 Jan 7;12:726719. doi: 10.3389/fphys.2021.726719 (PMC8777120; doi:10.3389/fphys.2021.726719)
Supplement: Supplementary file 1 [file Table_1.docx]

**Table S1 Multivariate regression analysis between function and structural**

**Model Summary**^b^

| Model | R | R Square | Adjusted R Square | Std. Error of the Estimate | Durbin-Watson |
| --- | --- | --- | --- | --- | --- |
| 1 | 0.893^a^ | 0.798 | 0.792 | 9.282 | 2.061 |

a. Variables: (Constant), LVMI, RWT.

b. Dependent variable: EDV

**Coefficients^a^**

| Variables | Standadizd Coeffcients | Standadized Coeffcients | 95% Confidence  interval | P value |
| --- | --- | --- | --- | --- |
| Constant | 125.423 |  |  | <0.001 |
| RWT | -263.671 | -.667 | (-0.435, -0.818) | <0.001 |
| LVMI | 0.776 | .813 | (0.624, 0.866) | <0.001 |
